# Epidemiology, risk factors, and spatio-temporal distribution of lumpy skin disease in cattle in northern Bangladesh

**DOI:** 10.5713/ab.260493

**Published:** 2026-09-16

**Authors:** Md. Shajedur Rahman, Md. Khaled Hossain, Jahangir Alam, Nazmi Ara Rumi, Md. Tanvir Islam, Md. Hasan Ali, Shihab Ahmed, Hemayet Hossain, Jong-Joo Kim, Md Azizul Haque

**Affiliations:** 1 Department of Microbiology, Hajee Mohammad Danesh Science and Technology University, Dinajpur, Bangladesh; 2 Department of Medicine, Surgery and Obstetrics, Hajee Mohammad Danesh Science and Technology University, Dinajpur, Bangladesh; 3 Animal Biotechnology Division, National Institute of Biotechnology, Dhaka, Bangladesh; 4 Department of Veterinary Biomedical Sciences, Lewyt College of Veterinary Medicine, Brookville, NY, USA; 5 Faculty of Veterinary, Animal and Biomedical Sciences, Sylhet Agricultural University, Sylhet, Bangladesh; 6 Antimicrobial Resistance Reference Laboratory (Research), Bangladesh Livestock Research Institute, Dhaka, Bangladesh; 7 Department of Anatomy and Histology, Sylhet Agricultural University, Sylhet, Bangladesh; 8 Faculty of Veterinary and Animal Science, Teesta University, Rangpur, Bangladesh; 9 Department of Biotechnology, Yeungnam University, Gyeongsan, Korea; 10 Department of Health Informatics, Faculty of Medicine and Health Sciences, Frontier University, Garowe Campus, Puntland, Somalia

**Keywords:** Bangladesh, Lumpy Skin Disease Virus, Molecular Epidemiology, Risk Factors, Seasonal Dynamics, Vector-borne Transmission

## Abstract

**Objective:**

Lumpy skin disease (LSD) is an emerging transboundary viral disease that leads to substantial economic losses in cattle production worldwide. Therefore, the present study aimed to investigate the epidemiology, risk factors, and spatial–temporal patterns through molecular detection of LSD virus among cattle in northern Bangladesh.

**Methods:**

A cross-sectional study was conducted from September 2023 to August 2025 across 10 districts involving 8,650 cattle from 1,200 households. Clinical examination, questionnaire-based epidemiological investigation, spatial mapping, and polymerase chain reaction targeting the *P32* and thymidine kinase genes were performed.

**Results:**

The prevalence of LSD was 30.15% (2,608/8,650; 95% confidence interval [CI]: 29.2–31.1), while the mortality and case fatality rates were 3.02% and 10.01%, respectively. Marked geographical and seasonal variations were observed, with the highest prevalence recorded in Thakurgaon (33.92%) and during the rainy season (44.56%). Calves less than one year had the highest prevalence (37.39%) and had an infection susceptibility about double that of older cattle (odds ratio [OR] = 2.04; 95% CI: 1.82–2.29). Unvaccinated cattle showed markedly higher prevalence (36.45%) of LSD. Multivariable logistic regression identified non-vaccination (adjusted odds ratio [AOR] = 14.3; 95% CI: 9.63–21.7), poor biosecurity (AOR = 5.18; 95% CI: 3.37–8.08), and large herd size (AOR = 2.80; 95% CI: 1.87–4.22) as major independent predictors of LSD prevalence.

**Conclusion:**

The current study demonstrates that LSD is widely distributed and strongly influenced by management, environmental, and host-related factors in northern Bangladesh. Strengthening vaccination coverage, farm biosecurity protocols, vector management strategies, and targeted surveillance systems may substantially reduce the burden of LSD in cattle populations.

## INTRODUCTION

Lumpy skin disease (LSD) is a highly contagious transboundary viral disease of cattle and buffalo caused by the LSD virus (LSDV), a member of the Poxviridae family of the genus Capripoxvirus [1,2]. Importantly, LSD is not a zoonotic disease and poses no direct risk to human health. The disease is characterized clinically by fever, enlarged superficial lymph nodes, circumscribed skin nodules, edema, nasal discharge, lacrimation, reduced milk production, infertility, emaciation, and occasionally death [3,4]. LSD imposes substantial economic losses on the livestock industry through reduced milk and meat production, poor hide quality, infertility, treatment costs, reduced draft power, and restrictions on national and international trade of animals and animal products [5,6]. Historically, the disease was restricted mainly to African countries; however, during the last decade, it has spread rapidly across the Middle East, Europe, and Asia, becoming one of the most important emerging transboundary animal diseases affecting cattle production worldwide [7,8]. The continuing expansion of LSD into previously disease-free regions highlights the urgent need for improved epidemiological surveillance, rapid diagnosis, and effective control strategies.

Bangladesh officially reported LSD outbreaks in cattle in 2019 [3]. Outbreaks have been reported from several districts, including Chattogram, Natore, Dinajpur, Rajshahi, Rangpur, and other northern regions of Bangladesh [3,9]. The northern region also supports one of the highest cattle densities in Bangladesh, facilitating close animal contact and increasing the likelihood of disease transmission. The tropical monsoon climate of Bangladesh, characterized by high temperature, humidity, and seasonal rainfall, provides favourable ecological conditions for blood-feeding arthropods such as mosquitoes, biting flies, and several tick species, all of which are recognized as important mechanical vectors of LSDV [7,10]. Increased vector abundance during warm and humid months substantially enhances virus transmission between susceptible cattle. Consequently, integrated vector management is considered an essential component of LSD prevention and control. Furthermore, frequent introduction of newly purchased cattle into existing herds without adequate quarantine, communal grazing practices, livestock trading, unrestricted animal movement, and inadequate farm biosecurity may facilitate virus introduction and persistence within cattle populations [6,11]. Despite the increasing occurrence of LSD outbreaks, comprehensive epidemiological studies focusing on the northern part of Bangladesh remain limited.

Several animal-level and farm-level factors have been associated with the occurrence and spread of LSD in both endemic and newly affected regions [5,6]. Age has been reported as an important determinant, with younger and immunologically naïve animals often showing increased susceptibility [11]. Breed-related differences have also been documented, with crossbred and exotic cattle generally exhibiting higher susceptibility than indigenous breeds [3,6]. Vaccination status plays a crucial role in disease prevention, and unvaccinated cattle are at significantly greater risk of infection [4,8]. In addition, large herd size, intensive farming systems, poor sanitation, inadequate biosecurity, and high vector density have been identified as important risk factors for LSD occurrence [5,11]. Seasonal variation further influences disease dynamics, as increased vector activity during warm and humid periods may accelerate virus transmission [10]. Understanding these epidemiological determinants is essential for designing evidence-based prevention and control programs. However, large-scale investigations evaluating these factors under field conditions in northern Bangladesh are still scarce.

Several epidemiological investigations have reported varying prevalence and disease burden of LSD in different regions of Bangladesh [3,12,13]. In the northwestern region of Bangladesh, Azam et al. [14] reported an overall attack rate of 37.6%, a mortality rate of 2.8%, and a case fatality rate (CFR) of 7.5% among affected cattle populations. The same study observed that calves younger than one year of age had higher mortality (4.1%) and CFR (10.0%) compared with adult cattle. In the Tanore and Puthia upazilas of Rajshahi district, Khan et al. [13] documented morbidity, mortality, and CFR of 71.42%, 7.14%, and 10%, respectively, indicating severe disease impact in some outbreak areas. Similarly, a study conducted in Chuadanga district reported an overall incidence risk of 22.24% during August–October 2022 [15]. These findings indicate considerable variation in the prevalence and severity of LSD across different regions of Bangladesh, which may be influenced by geographical location, climatic conditions, cattle density, management practices, vaccination coverage, and vector abundance. Therefore, comprehensive epidemiological studies covering multiple districts are necessary to better understand the disease dynamics and burden of LSD in Bangladesh.

Accurate laboratory diagnosis is essential for effective surveillance and control of LSD [9]. Polymerase chain reaction (PCR) is currently considered the gold standard method for molecular detection of LSDV owing to its high sensitivity and specificity [16,17]. PCR assays targeting genes such as *P32, TK, GPCR*, and RPO30 have been successfully used for the detection and molecular characterization of LSDV strains worldwide [9,18]. Molecular diagnosis not only confirms clinical cases but also provides valuable information regarding viral circulation and epidemiology [19].

In addition to laboratory confirmation, spatial and temporal epidemiological analyses are increasingly being used to understand disease dynamics and transmission patterns [20,21]. Previous studies in Uganda demonstrated significant spatial clustering of LSD outbreaks and highlighted the role of cattle movement and environmental factors in disease spread [20]. Similar investigations in Thailand reported distinct spatiotemporal patterns associated with climatic conditions and vector abundance [21]. In India, geospatial analyses revealed hotspot regions with increased outbreak frequency and emphasized the importance of targeted surveillance and intervention measures [22]. Recent studies have also shown that rainfall, humidity, and temperature significantly influence the temporal distribution of LSD outbreaks by affecting vector populations and viral transmission dynamics [23]. Spatial mapping and hotspot analysis can therefore facilitate identification of high-risk areas and improve disease control planning. Nevertheless, integrated studies combining prevalence estimation, risk factor analysis, spatial–temporal epidemiology, and molecular detection of LSD across multiple districts of northern Bangladesh remain limited.

Considering the increasing occurrence of LSD and the lack of comprehensive epidemiological and molecular information from northern Bangladesh, the present study was undertaken to investigate the prevalence, spatial–temporal distribution, associated risk factors, and molecular detection of LSD among cattle in selected districts of northern Bangladesh. The findings of this study are expected to contribute to a better understanding of LSD epidemiology and support the development of evidence-based prevention and control strategies for cattle populations in Bangladesh.

## MATERIALS AND METHODS

### Study area and study period

The study was conducted in 10 upazilas under 10 districts of the northern part of Bangladesh over two years from September 2023 to August 2025 (Figure 1). The selected districts represented major cattle-rearing areas with varying agroecological and management conditions. According to the Department of Livestock Services, these districts collectively contain a substantial proportion of the cattle population of northern Bangladesh, making them suitable for epidemiological investigation of LSD.

### Study population and sample size

The study population comprised cattle reared in selected households within the study areas, irrespective of age, sex, breed, and production purpose. Both indigenous and crossbred cattle managed under intensive and semi-intensive production systems were included. In this study, we did not find free range rearing of cattle during the study period. A total of 1,200 cattle-rearing households were surveyed, comprising 8,650 cattle, all of which underwent clinical examination for evidence of LSD.

### Study design and sampling strategy

A cross-sectional epidemiological study was conducted in the northern part of Bangladesh. A multistage sampling approach was employed for the selection of study areas, households, and animals. Districts were selected based on cattle population density, previous reports of LSD outbreaks, accessibility, and the importance of cattle farming in the local economy. Upazilas were selected from each district based on livestock population, history of LSD occurrence reported by the Department of Livestock Services, and consultation with local veterinary authorities. Finally, cattle-rearing households were selected through systematic random sampling from lists obtained from local livestock offices and community livestock workers. Where updated household lists were unavailable, households were selected through farm-to-farm visits using a structured sampling interval.

### Sample size determination

The minimum required sample size for estimation of disease prevalence was calculated using the standard formula for cross-sectional studies:


(1) $$\text{n}=\frac{{\text{Z}}^{2}\text{P}\times (1-\text{P})}{{\text{d}}^{2}}$$

where n = required sample size, Z = 1.96 at 95% confidence level, P = expected prevalence, and d = desired absolute precision.

Assuming an expected prevalence of 50%, a confidence level of 95%, and a precision of 5%, the minimum calculated sample size was 384 cattle. However, to improve the precision of prevalence estimates and to allow robust risk factor analysis across multiple districts, a substantially larger sample comprising 8,650 cattle from 1,200 households was included in the study.

All cattle present in the selected households at the time of the survey were included in the clinical investigation following molecular diagnosis of 400 representative clinically suspected case by PCR.

### Questionnaire survey and epidemiological data collection

A structured questionnaire was developed based on published literature and expert consultation. The questionnaire was designed to obtain information on: animal characteristics, herd management, vaccination, production system, animal movement, housing, biosecurity, vector abundance and clinical history. Herd size was classified as: small (1–5 cattle), medium (6–10 cattle) and large (11 or more cattle). Farm biosecurity was evaluated using a structured checklist comprising: isolation of clinically sick animals, routine cleaning and disinfection, use of footbaths, separate feeding and watering equipment for sick cattle and implementation of vector control measures. Information regarding the introduction of newly purchased cattle into the herd was collected and evaluated as a potential risk factor. Vector density was assessed during farm visits based on the observed and reported abundance of mosquitoes, biting flies, and ticks around cattle housing. Clinical examination findings and epidemiological information were recorded simultaneously using a standardized data collection form.

### Clinical examination and case definition

All cattle included in the study were subjected to physical and clinical examination following the guidelines described in the World Organisation for Animal Health (WOAH) Terrestrial Manual for Lumpy Skin Disease and previously published reports on LSD outbreaks [3,4,17]. Clinical diagnosis of LSD was based on the presence of characteristic signs including fever, enlarged superficial lymph nodes, circumscribed skin nodules, edema of limbs and brisket, lacrimation, nasal discharge, reduced milk production, anorexia, and weakness [17,19]. Particular attention was given to the presence of generalized skin nodules distributed over the neck, trunk, perineum, udder, and limbs, which are considered hallmark lesions of LSD. A suspected clinical case of LSD was defined as any cattle exhibiting one or more characteristic skin nodules with or without accompanying systemic signs such as fever, lymphadenopathy, edema, ocular discharge, or nasal discharge, according to the case definition described by WOAH and Tuppurainen et al. [4]. Animals presenting typical generalized nodular lesions were considered clinically positive and selected for further epidemiological investigation and laboratory confirmation.

### Sample collection and transportation

Clinical samples were collected aseptically from cattle showing characteristic signs of LSD for molecular confirmation. The collected samples included: skin lesion tissue and swab, scab materials, whole blood, nasal swabs, and saliva. Samples were collected using sterile instruments and transferred into sterile screw-capped viral transport media (VTM). Blood samples were collected in EDTA-containing vacutainer tubes. Each sample was labelled with animal identification number, district, upazila, sample type, and date of collection. Following collection, samples were immediately preserved in ice boxes containing frozen gel packs and transported to the laboratory under cold chain conditions. Samples were subsequently stored at −20°C until laboratory processing.

### DNA extraction

Viral DNA was extracted from clinical samples using a commercially available DNA extraction kit, QIAamp DNA Mini Kit (Qiagen), according to the manufacturer’s instructions. Briefly, skin tissue and scab samples were homogenized using a sterile mortar and pestle or tissue homogenizer before extraction. Approximately 200 μL of blood or homogenized tissue suspension was processed for DNA extraction. The extracted DNA was eluted in elution buffer and stored at −20°C until PCR amplification.

### Polymerase chain reaction

PCR amplification targeting the *P32* and *thymidine kinase (TK)* genes of LSDV was performed for molecular confirmation of representative clinically suspected samples (Figure 2). The *P32* gene was selected due to its conserved nature and widespread use in routine LSDV diagnosis, while the *TK* gene was employed as an additional confirmatory molecular marker.

### Primers

Specific primers targeting the *P32* and *TK* genes were used according to previously published protocols (Table 1) [24,25].

### Polymerase chain reaction mixture

PCR amplification was performed in a final reaction volume of 25 μL containing 12.5 μL PCR master mix, 1 μL forward primer, 1 μL reverse primer, 5 μL template DNA, and 5.5 μL nuclease-free water.

### Polymerase chain reaction cycling conditions

PCR reactions were performed using a T100 Thermal Cycler (Bio-Rad) under optimized conditions specific for each primer set. For the *P32* gene, the amplification protocol consisted of an initial denaturation at 95°C for 1 minute, followed by 30 cycles of denaturation at 95°C for 30 seconds, annealing at 58°C for 30 seconds, and extension at 72°C for 30 seconds, with a final extension at 72°C for 5 minutes. For the *TK* gene, the thermal cycling conditions included an initial denaturation at 95°C for 1 minute, followed by 35 cycles of denaturation at 95°C for 45 seconds, annealing at 56°C for 45 seconds, and extension at 72°C for 1 minute, with a final extension step at 72°C for 7 minutes. All reaction conditions were optimized to ensure specific amplification of the target genes.

### Agarose gel electrophoresis

Amplified PCR products were separated by electrophoresis on a 1.5% agarose gel stained with ethidium bromide. Electrophoresis was carried out at 100 volts for approximately 45–60 minutes. DNA bands were visualized under ultraviolet illumination using a gel documentation system and compared with a molecular weight DNA ladder.

### Spatial and temporal distribution

Spatial distribution of LSD cases was depicted using Geographic Information System (GIS) software (ArcMap 10.8). Geographic coordinates recorded from affected households were used to generate spatial prevalence maps across the study areas. Temporal distribution analysis was performed using monthly and seasonal outbreak data collected during the study period. Seasonal occurrence patterns were evaluated according to summer, rainy, autumn, and winter seasons to determine temporal variation in disease incidence. Monthly mean prevalence was computed for each district by averaging the prevalence values across the two years. Standard errors (SE) of the monthly means were calculated using the formula:


(2) $$\text{SE}=\sqrt{\frac{\sum {\text{SE}}_{\text{i}}^{2}}{\text{n}}}$$

where ${\text{SE}}_{\text{i}}=\sqrt{\frac{{\text{p}}_{\text{i}}\left(1-{\text{p}}_{\text{i}}\right)}{{\text{N}}_{\text{i}}}}$, P_i_ is the prevalence for a given year, *N_i_* is the total examined, and *n* is the number of years. This approach accounts for variability across years and ensures accurate estimation of uncertainty.

### Visualization

District-wise monthly mean prevalence and SE were visualized using line plots with circular markers representing mean prevalence, a dark blue ribbon indicating SE, and a dotted trend line overlay to highlight temporal patterns. Seasonal variations were annotated with background shading for Winter (Dec–Feb), Summer (Mar–May), Rainy (Jun–Aug), and Autumn (Sep–Nov) to facilitate interpretation of seasonal trends.

### Statistical analysis

Statistical analyses were conducted in R (ver. 4.4.3) using the gtsummary package to ensure reproducibility and transparency of the results. Only questionnaires with complete responses for all items were included in the final analysis. Descriptive statistics were summarized as frequencies and percentages for categorical variables. Pearson’s chi-square test was used to assess differences in disease prevalence among categories of explanatory variables. Univariable and multivariable logistic regression analyses were performed to evaluate the associations between independent variables and the disease outcome. Variables with a p-value<0.20 in the univariable analysis were considered eligible for inclusion in the multivariable logistic regression model. Adjusted odds ratios (AORs) with 95% confidence intervals (95% CIs) were estimated for each explanatory variable, and statistical significance was considered at p<0.05.

## RESULTS

Clinical manifestations of LSD varied among the affected cattle during the study period. Representative clinical signs and lesion progression observed in clinically infected animals are presented in Figure 3. A total of 8,650 cattle from 10 districts of northern Bangladesh were investigated over a 24-month surveillance period, of which 2,608 animals were clinically diagnosed as LSD, yielding an overall prevalence of 30.15% (95% CI: 29.2–31.1). The overall mortality and CFR were 3.02% (261/8,650; 95% CI: 2.7–3.4) and 10.01% (261/2,608; 95% CI: 8.9–11.2), respectively (Supplement 1; Figure 4). PCR analysis of representative clinically suspected samples confirmed the presence of LSDV by successful amplification of the *P32* and *TK* genes, thereby supporting the clinical diagnosis of LSD.

### Spatial distribution of lumpy skin disease

Considerable geographical variation in LSD prevalence was observed among the studied districts (χ^2^ = 26.23, p = 0.0019) (Table 2). The highest prevalence was recorded in Thakurgaon (33.92%), followed by Nilphamari (33.22%), Kurigram (31.62%), Dinajpur (31.35%), and Joypurhat (30.47%) (Table 2). In contrast, the lowest prevalence was documented in Bogura (25.44%). Compared with Bogura, cattle from Thakurgaon had significantly higher odds of infection (OR = 1.50; 95% CI: 1.22–1.86; p<0.001), followed by Nilphamari (OR = 1.46; 95% CI: 1.18–1.80; p<0.001), Kurigram (OR = 1.36; 95% CI: 1.09–1.68; p = 0.006), and Dinajpur (OR = 1.34; 95% CI: 1.08–1.66; p = 0.007). Mortality rates varied from 2.40% in Rangpur to 3.41% in Dinajpur, while CFR ranged from 8.47% in Rangpur to 10.89% in Bogura.

### Seasonal and temporal distribution

Marked seasonal variation in LSD occurrence was detected (p<0.001). The rainy season exhibited the highest prevalence (44.56%), mortality (5.60%), and CFR (12.58%), followed by autumn (34.07%), summer (30.75%), and winter (10.65%). Relative to winter, cattle were significantly more likely to be infected during the rainy season (OR = 6.74; 95% CI: 5.75–7.94; p<0.001), autumn (OR = 4.34; 95% CI: 3.69–5.11; p<0.001), and summer (OR = 3.73; 95% CI: 3.16–4.40; p< 0.001). District-wise analysis revealed marked seasonal fluctuations in LSD prevalence. Peak mean prevalence was observed during the Rainy season (June–August) across most districts, reaching up to 52.6% in Thakurgaon (August), followed by 51.9% in Thakurgaon (June) and 51.3% in Thakurgaon (July). In contrast, the lowest prevalence was recorded during Winter months (December–February), with mean values as low as 4.74% in Bogura (February), 4.74% in Gaibandha (February), and 6.17% in Gaibandha (December).

### District-level variation

Among the districts, Thakurgaon exhibited the highest peak mean prevalence during the rainy season, whereas Gaibandha and Bogura maintained comparatively lower prevalence levels. The SE ribbons indicated moderate inter-annual variability, with higher uncertainty in some districts during transitional months.

### Seasonal trends

The data demonstrated a clear seasonal trend, with Summer months showing a gradual increase in prevalence, peaking during the Rainy season, followed by a decline during Autumn, and reaching a minimum in Winter (Figures 5, 6). This pattern was consistent across all districts, highlighting the influence of seasonal climatic factors on LSD outbreaks.

Monthly analysis further demonstrated recurrent epidemic peaks during the monsoon period. The highest prevalence was recorded in August 2024 (46.76%), followed by July 2024 (45.30%), August 2025 (44.68%), and July 2025 (44.41%). Conversely, the lowest prevalence was observed during the winter months, particularly February 2025 (7.87%), February 2024 (8.93%), and January 2024 (9.27%). Mortality and CFR similarly increased during the rainy months, with the highest mortality observed in July 2024 (6.35%) and the highest CFR in September 2024 (15.67%).

### Association of host-related factors with lumpy skin disease occurrence

Significant associations were observed between LSD occurrence and host-related factors including age, breed, and sex. Age was strongly associated with disease occurrence (χ^2^ = 156.86, p<0.001) (Table 3). Calves aged below one year showed the highest prevalence (37.39%), mortality (5.98%), and CFR (15.99%), and were approximately two times more likely to be infected compared with adult cattle (>3 years) (OR = 2.04; 95% CI: 1.82–2.29; p<0.001). Young cattle aged 1–3 years also demonstrated significantly higher odds of infection (OR = 1.44; 95% CI: 1.28–1.63; p<0.001).

Breed-wise analysis revealed significant differences in susceptibility (χ^2^ = 74.47, p<0.001). Sahiwal crossbred cattle had the highest prevalence (37.02%), mortality (4.38%), and CFR (11.83%), with significantly greater odds of infection compared with local breeds (OR = 1.59; 95% CI: 1.43–1.77; p< 0.001). Although Friesian crossbred cattle showed slightly higher prevalence (28.87%) than local breeds (27.03%), the difference was not statistically significant (p = 0.136).

Sex also showed a statistically significant association with LSD prevalence (χ^2^ = 4.50, p = 0.034). Female cattle exhibited higher prevalence (31.14%) and mortality (3.27%) than males (29.02% and 2.73%, respectively), with females having 1.11 times higher odds of infection (95% CI: 1.01–1.21; p = 0.032).

### Association of management and environmental factors with lumpy skin disease occurrence

Vaccination status demonstrated a profound effect on LSD occurrence (p<0.001). Non-vaccinated cattle showed substantially higher prevalence (36.45%) compared with vaccinated animals (9.79%) (Table 4). Likewise, mortality and CFR were markedly higher among non-vaccinated cattle (3.81% and 10.47%, respectively) than vaccinated cattle (0.44% and 4.50%, respectively). Non-vaccinated animals were more than five times more likely to develop LSD (OR = 5.28; 95% CI: 4.54–6.18; p<0.001).

Herd size was significantly associated with disease occurrence (χ^2^ = 27.16, p<0.001). Large herds (11 or more cattle) showed the highest prevalence (32.08%), while medium herds (6–10 cattle) exhibited the highest mortality (3.81%) and CFR (12.18%). Compared with small herds, cattle from medium and large herds had significantly higher odds of infection, with OR values of 1.31 (95% CI: 1.16–1.48; p<0.001) and 1.36 (95% CI: 1.20–1.54; p<0.001), respectively (Table 4).

The production system also significantly influenced disease prevalence (χ^2^ = 96.01, p<0.001). Semi-intensive farms exhibited significantly higher prevalence (34.86%) compared with intensive systems (25.17%), with cattle under semi-intensive management showing 1.59 times higher odds of infection (95% CI: 1.45–1.75; p<0.001).

Biosecurity status demonstrated a strong association with LSD occurrence (χ^2^ = 246.70, p<0.001). Farms with poor biosecurity practices exhibited the highest prevalence (37.72%), mortality (4.93%), and CFR (13.08%). Compared with farms maintaining good biosecurity, cattle from farms with moderate and poor biosecurity had significantly elevated odds of infection, with OR values of 2.20 (95% CI: 1.93–2.51; p<0.001) and 2.88 (95% CI: 2.52–3.31; p<0.001), respectively.

Similarly, vector density was strongly associated with LSD prevalence (χ^2^ = 444.67, p<0.001). Herds with high vector density exhibited the highest prevalence (41.81%), mortality (4.92%), and CFR (11.77%). Compared with low vector density herds, cattle from medium- and high-vector-density environments had significantly increased odds of infection, with OR values of 2.35 (95% CI: 2.05–2.68; p<0.001) and 3.93 (95% CI: 3.44–4.48; p<0.001), respectively.

### Logistic regression analysis of farm-level risk factors

Multivariate logistic regression analysis identified several independent predictors significantly associated with LSD occurrence at the farm level. Non-vaccinated farms had the strongest association with disease occurrence, showing 14.3 times higher odds of LSD compared with vaccinated farms (AOR = 14.3; 95% CI: 9.63–21.7; p<0.001) (Table 5).

Large herd size remained a significant predictor after adjustment, with large farms exhibiting 2.80 times higher odds of LSD occurrence than small farms (AOR = 2.80; 95% CI: 1.87–4.22; p<0.001), while medium-sized farms had 1.76 times higher odds (AOR = 1.76; 95% CI: 1.28–2.43; p<0.001). Poor biosecurity was another major determinant, increasing the odds of LSD by more than fivefold (AOR = 5.18; 95% CI: 3.37–8.08; p<0.001), whereas moderate biosecurity increased the odds nearly twofold (AOR = 1.96; 95% CI: 1.32–2.92; p<0.001).

Seasonal month-wise regression analysis showed significantly increased risk during June (AOR = 7.67; 95% CI: 3.45–17.4; p<0.001), July (AOR = 2.70; 95% CI: 1.29–5.71; p = 0.009), and November (AOR = 2.45; 95% CI: 1.21–5.01; p = 0.013), compared with April. In contrast, January and February were associated with significantly reduced odds of disease occurrence (AOR = 0.32 for both months; p = 0.003).

## DISCUSSION

Lumpy skin disease is an economically devastating infectious disease of cattle that represents a major challenge to livestock health and sustainable cattle production worldwide [26]. In Bangladesh, the disease continues to be reported every year. It imposes a substantial economic burden on livestock farmers through reduced milk production, impaired growth performance, reproductive losses, treatment costs, and mortality [27]. Although several studies have reported the prevalence of LSD in Bangladesh, comprehensive investigations addressing associated risk factors and epidemiological determinants remain limited [3,13,28]. Therefore, the present study provides a comprehensive epidemiological assessment of LSD among cattle in northern Bangladesh over a 24-month surveillance period and highlights substantial spatial, seasonal, host-related, and management-associated variation in disease occurrence.

The overall prevalence of LSD recorded in the present study was 30.15%, which was slightly lower than the 41.06% previously reported in cattle in Dinajpur Sadar, Bangladesh [29], and the 49.01% documented in Mymensingh [28], but higher than the 24.59% national seroprevalence previously reported in Bangladesh [30] and broadly comparable to the 33.44% recently documented in northern Bangladesh [31]. These variations may be attributed to differences in geographical coverage, surveillance period, outbreak intensity, and farm-level management and disease control practices. The mortality rate observed in the present study (3.02%) was slightly lower than the 7.14% reported in cattle from Tanore and Puthia upazilas in Rajshahi, Bangladesh [13]. In comparison the CFR (10.01%) was comparable to the 10% previously documented in the same regions. These differences may be attributed to variations in outbreak severity, vaccination coverage, and farm-level management practices, ultimately contributing to differing levels of economic loss at the farm level.

Marked spatial variation in LSD prevalence was observed across districts, with the highest prevalence recorded in Thakurgaon (33.92%) and the lowest in Bogura (25.44%). The increased odds of infection in Thakurgaon (OR = 1.50) and neighbouring districts suggest localized transmission hotspots. These spatial differences likely reflect variations in cattle density, vector abundance, and implementation of control measures, indicating that LSD transmission is spatially clustered rather than uniformly distributed. A strong seasonal pattern was observed, with higher prevalence in the rainy season (44.56%) than in winter (10.65%). Infection risk was significantly elevated during the rainy season (OR = 6.74), with consistent peaks in July–August, consistent with previously established studies [3]. These findings highlight the key role of rainfall and humidity in enhancing vector abundance and driving LSD transmission. Host-related factors significantly influenced LSD occurrence. Calves below one year of age showed approximately twice the risk of infection compared to adults, likely reflecting immature immune responses and reduced ability to control systemic infection. Similar findings have been reported in Ethiopia [32]. Crossbred cattle exhibited higher prevalence [33], while females also showed slightly increased prevalence, possibly due to longer exposure within production systems. Similar findings have been reported in Uganda, where females were approximately twice as likely to be seropositive for LSD [34].

Management-related factors played a critical role in LSD dynamics. Vaccination emerged as the strongest protective factor against LSD, with significantly reduced infection risk observed in vaccinated cattle, underscoring the critical importance of sustained and high-coverage vaccination programs in limiting transmission, as consistently reported in previous epidemiological studies and international guidelines [35]. Herd size and production system significantly influenced infection risk, with larger herds and semi-intensive systems showing higher prevalence, likely due to increased animal contact and greater exposure to vectors in outdoor environments. Biosecurity and vector density emerged as key determinants of LSD occurrence. Poor biosecurity was associated with the highest prevalence (37.72%) and nearly threefold increased risk of infection, while high vector density substantially elevated prevalence (41.81%) and infection risk [36]. These findings emphasize that effective disease control requires strengthened farm biosecurity alongside integrated vector management strategies to interrupt transmission dynamics.

The study was limited by its cross-sectional design, which could not establish causal relationships between risk factors and disease occurrence. PCR confirmation was performed only on representative clinically suspected cases; therefore, subclinical infections may not have been detected. Molecular sequencing of LSDV strains and entomological investigation of vectors were not performed. Vector density was estimated using field observation and questionnaire responses rather than direct entomological measurement. In addition, some epidemiological data relied on farmer responses, which may have introduced self-reported bias.

Therefore, LSD in northern Bangladesh shows a clear endemic and seasonally driven pattern, with transmission strongly influenced by environmental conditions, host susceptibility, and management practices. The observed prevalence and case fatality indicate a persistent threat to cattle health and productivity, contributing to substantial economic losses for livestock farmers through reduced production efficiency, treatment costs, and animal losses [5]. The clear associations with vaccination status, biosecurity level, and vector density highlight that LSD is a preventable but management-dependent disease. Therefore, effective control requires an integrated approach combining sustained vaccination programs, improved biosecurity practices, and targeted vector control, particularly during high-risk rainy seasons [37]. Strengthening farmer awareness and implementing region-specific surveillance systems are also essential to reduce transmission and support early outbreak detection. Furthermore, the adoption of precision livestock farming technologies, including AI-based monitoring systems, may strengthen disease surveillance, facilitate early detection, and improve the effectiveness of LSD control programs [38–40].

## CONCLUSION

The present study provides a comprehensive epidemiological and molecular investigation of LSD in cattle across northern Bangladesh and demonstrates that LSD remains a significant endemic threat to cattle health and productivity in the region. The study identified considerable spatial and seasonal variation in disease occurrence, with increased prevalence during the rainy season and in districts characterized by higher vector density and intensive cattle production activities. Several important host-related and management-associated risk factors, including younger age, crossbred cattle, non-vaccination, large herd size, poor biosecurity, and semi-intensive farming systems, were significantly associated with increased disease occurrence. Molecular confirmation by PCR further verified the active circulation of LSDV among clinically affected cattle. The findings emphasize that effective control of LSD requires an integrated prevention strategy combining regular vaccination, strengthened farm biosecurity, improved vector control, and continuous epidemiological surveillance. In addition, farmer awareness and region-specific disease monitoring programs are essential to reduce transmission and minimize economic losses. These findings will contribute valuable baseline information for policymakers, veterinarians, and livestock authorities in developing evidence-based LSD prevention and control strategies in Bangladesh.

## Figures and Tables

**Figure 1:**
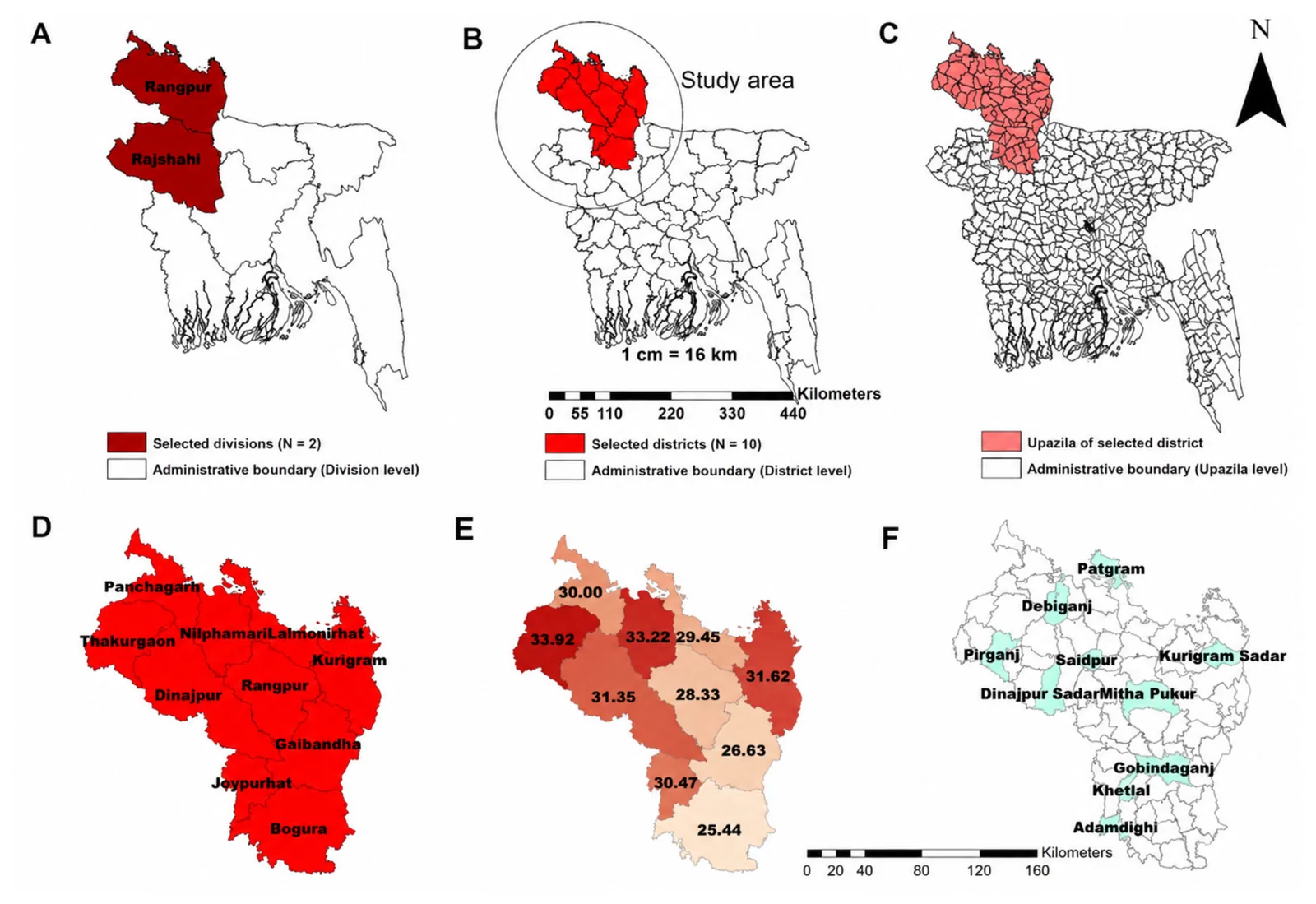
Study area and spatial distribution of lumpy skin disease (LSD) prevalence in northern Bangladesh. (A) Selected divisions, (B) selected districts within the study area, (C) selected upazilas, (D) study districts included in the survey, (E) district-wise prevalence (%) of LSD, and (F) locations of the selected upazilas. The map was prepared using ArcMap 10.8.

**Figure 2:**
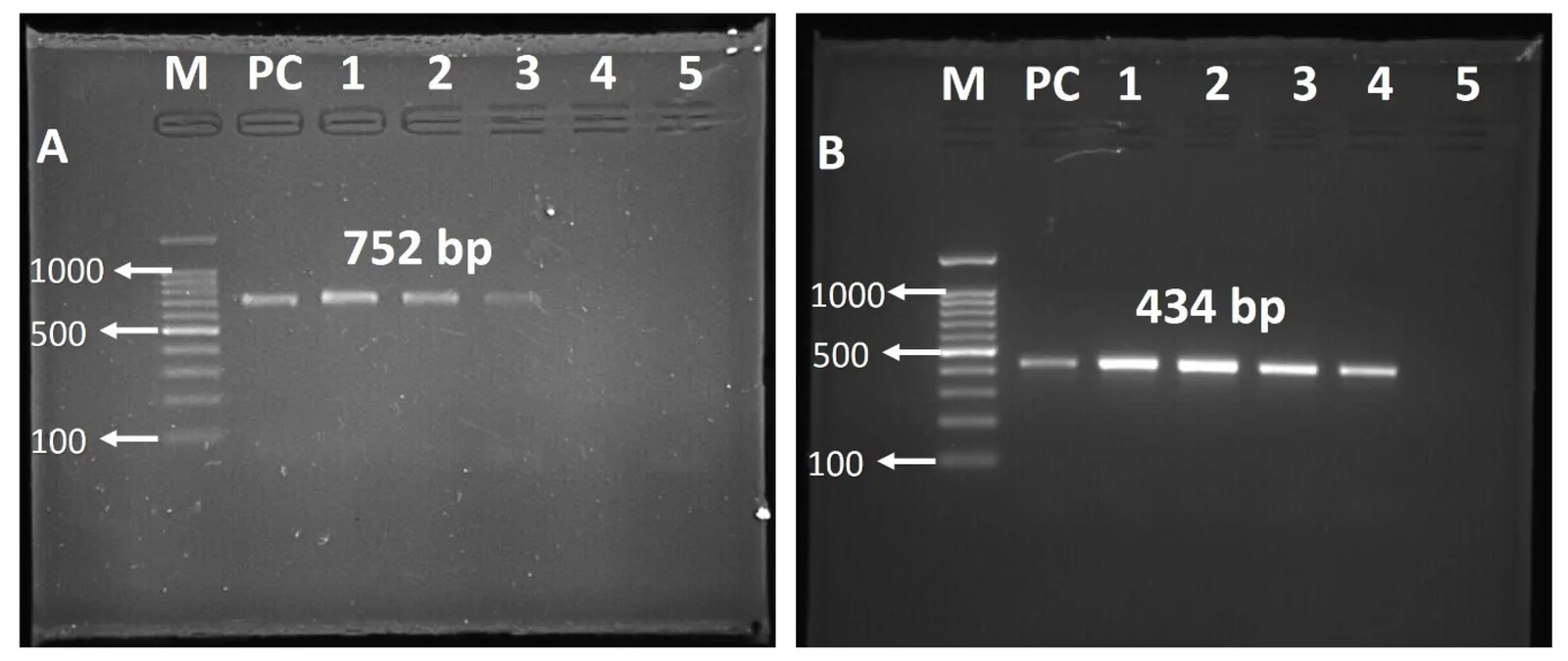
PCR amplification targeting the *P32* and *thymidine kinase* (*TK*) genes of LSDV, and their positive bands were shown at 752 bp (A), 434 bp (B), respectively. M, ladder DNA markers (100 bp DNA marker); PC, positive control; PCR, polymerase chain reaction; LSDV, lumpy skin disease virus.

**Figure 3:**
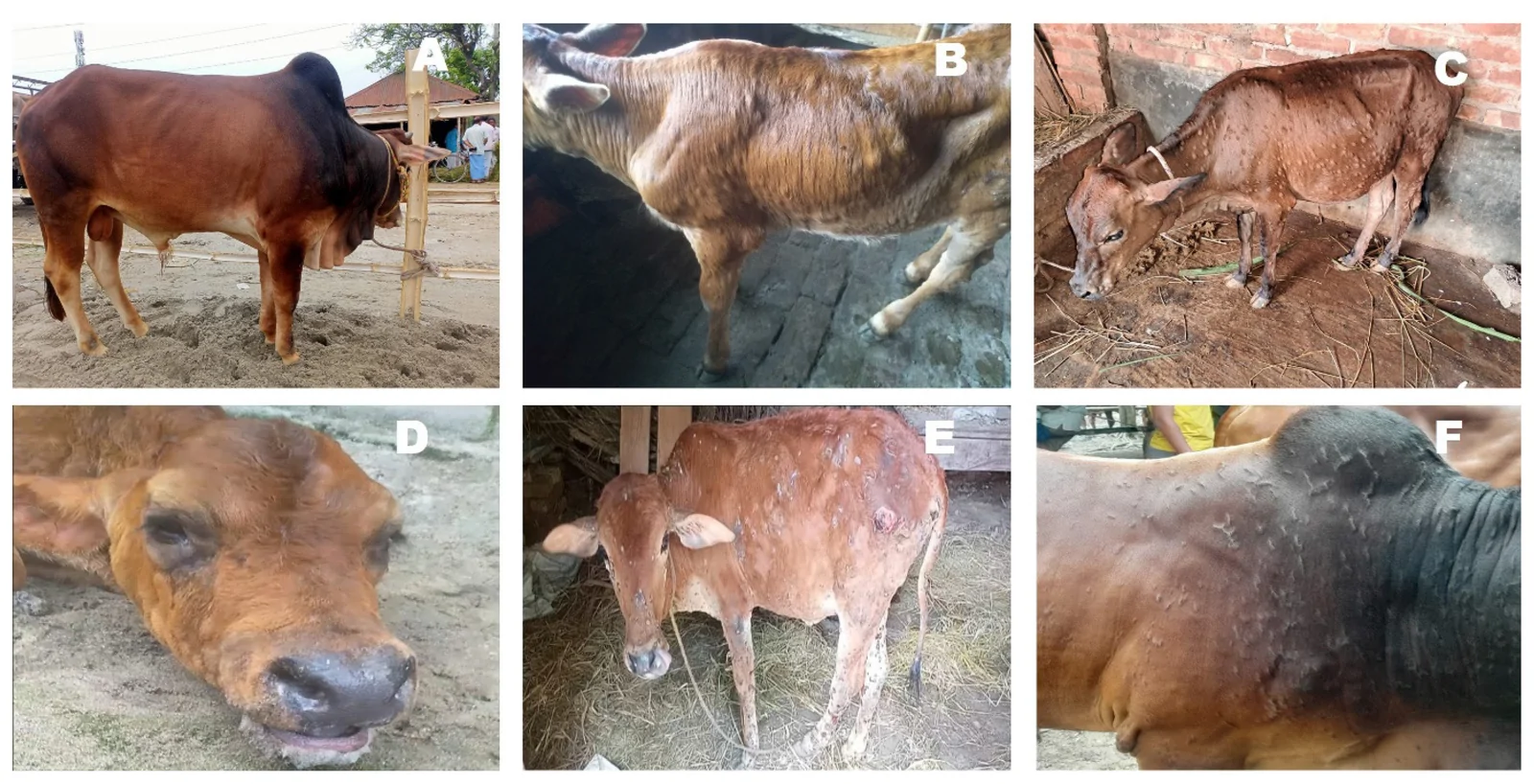
Representative clinical signs of lumpy skin disease (LSD) observed in cattle during the study period. (A) Healthy control animal. (B) Initial development of characteristic skin nodules. (C) Severe generalized nodular lesions distributed across the body surface. (D) Clinical complications including lacrimation, facial swelling, and respiratory distress. (E) Healing phase with necrosis, scab formation, and regression of skin lesions. (F) Healed lesion showing residual scar formation following recovery from LSD infection.

**Figure 4:**
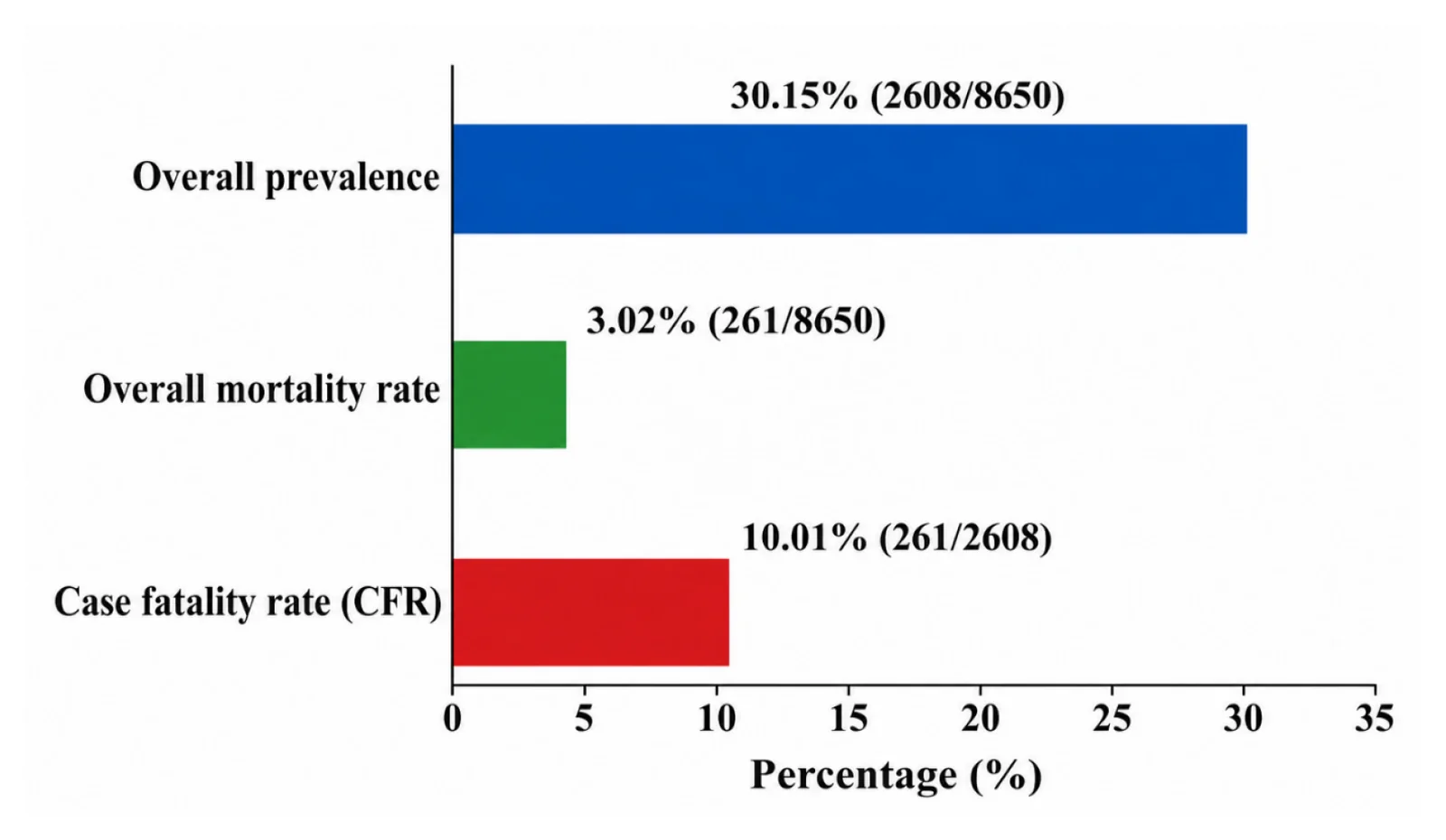
Horizontal bar chart showing the overall prevalence, mortality rate, and CFR of lumpy skin disease (LSD) among cattle in northern Bangladesh during the 24-month surveillance period.

**Figure 5:**
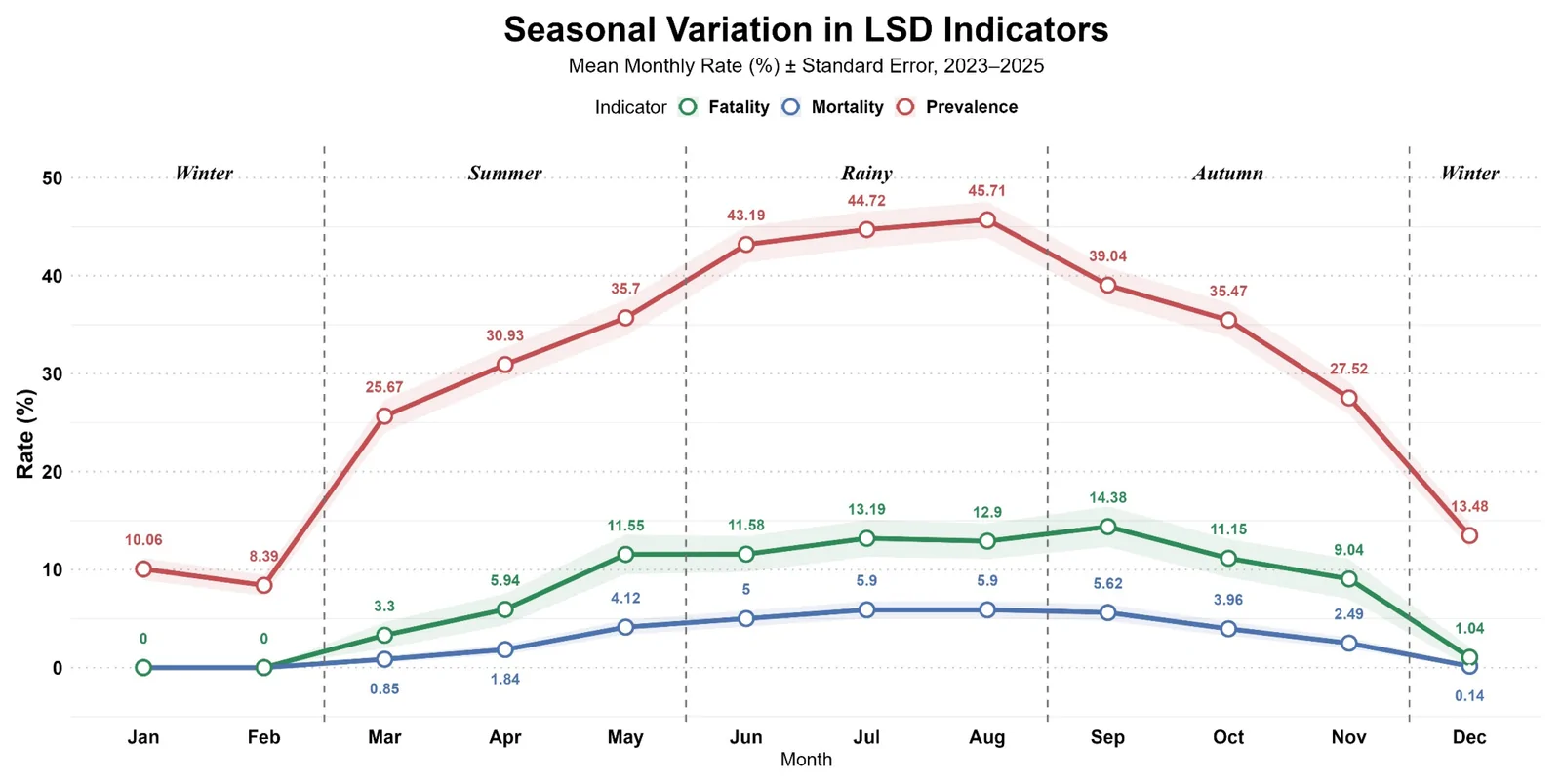
The seasonal variation of LSD prevalence, mortality, and fatality among the cattle in Northern Bangladesh. LSD, lumpy skin disease.

**Figure 6:**
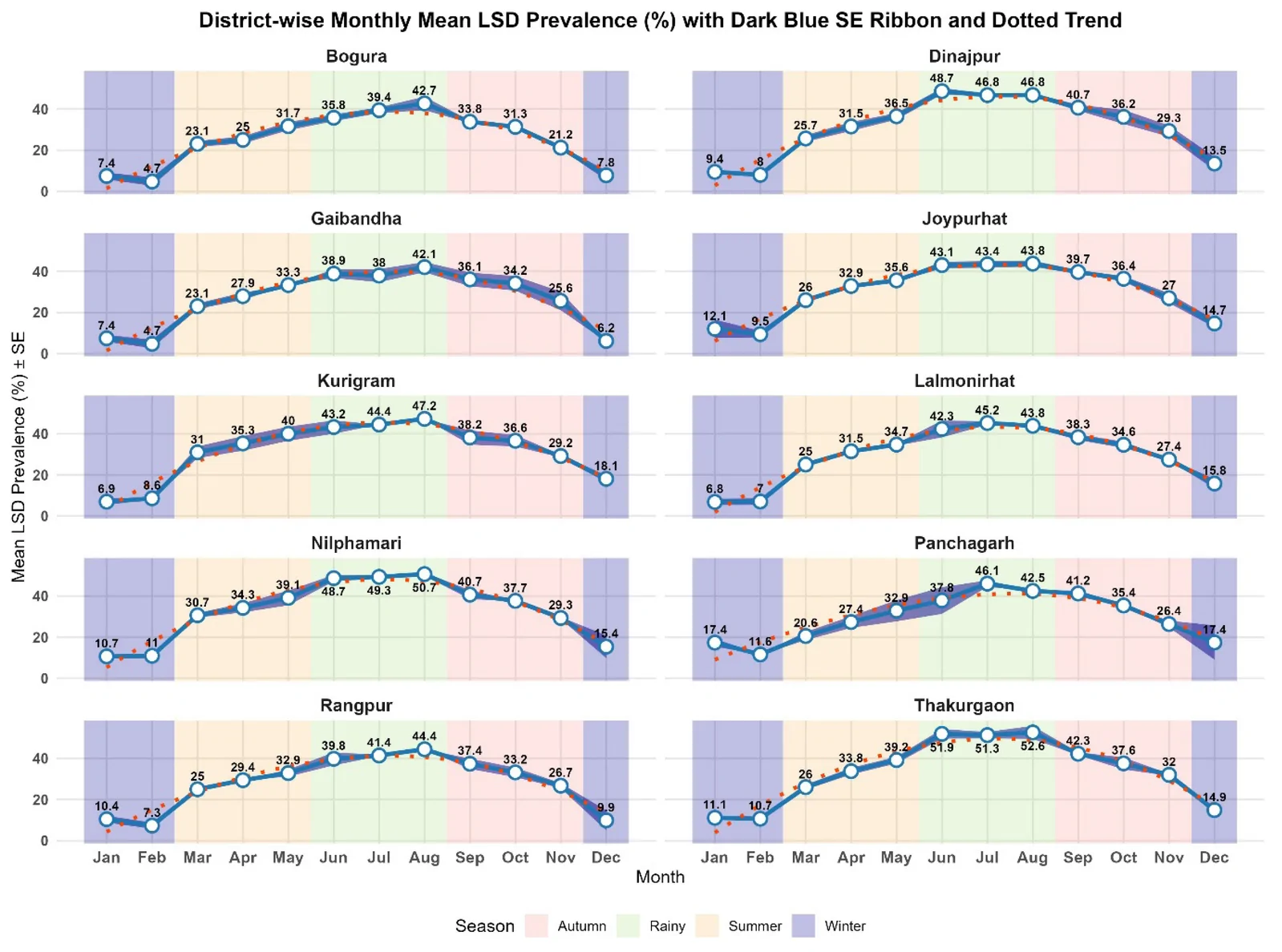
District-wise seasonal trends monthly mean prevalence (%) from September 2023 to August 2025 of lumpy skin disease with standard error (SE). Line plots depict the mean monthly prevalence (%) of lumpy skin disease in cattle across ten districts. Circular markers indicate mean prevalence, while the dark blue ribbons represent the SE across the two study years (2023–2025). The red dotted line highlights the overall monthly trend. Background shading denotes seasons: Winter (purple, Dec–Feb), Summer (orange, Mar–May), Rainy (green, Jun–Aug), and Autumn (pink, Sep–Nov). Peaks in prevalence during the Rainy season and minima during Winter are clearly observed across districts, indicating strong seasonal dynamics.

**Table 1:** Primers used in this study for amplification of a 752 bp genomic fragment which is specific to *P32* antigen gene and 434 bp genomic fragment, which is specific to thymidine kinase (*TK*) gene

Primer name	Primer sequence (5′ to 3′)	Amplicon size (bp)	Annealing temp. (°C)	Reference
P32-F	CGCGAAATTTCAGATGTAGTTCCA	752	58	[25]
P32-R	TGAGCCATCCATTTTCCAACTC			
DW-TKF	GCCGATAACATATATAGACCC	434	56	[24]
OP49-R	GTGCTATCTAGTGCAGCTAT			

**Table 2:** Spatial and seasonal distribution, prevalence, mortality, case fatality, and associated risk of lumpy skin disease in cattle in northern Bangladesh

	Positive/total examined	Prevalence (%)	Mortality (%)	CFR (%)	OR (95% CI)	p-value	χ^2^-value (df)	p-value
District								
Bogura	202/794	25.44	2.77	10.89	Ref.	-	26.23 (9)	0.0019
Panchagarh	270/900	30.00	3.22	10.74	1.26 (1.01–1.56)	0.037		
Thakurgaon	307/905	33.92	3.09	9.12	1.50 (1.22–1.86)	<0.001		
Dinajpur	285/909	31.35	3.41	10.88	1.34 (1.08–1.66)	0.007		
Lalmonirhat	255/866	29.45	2.89	9.80	1.22 (0.99–1.52)	0.068		
Nilphamari	297/894	33.22	3.13	9.43	1.46 (1.18–1.80)	<0.001		
Kurigram	271/857	31.62	3.15	9.96	1.36 (1.09–1.68)	0.006		
Rangpur	236/833	28.33	2.40	8.47	1.16 (0.93–1.44)	0.189		
Gaibandha	212/796	26.63	2.89	10.85	1.06 (0.85–1.33)	0.588		
Joypurhat	273/896	30.47	3.13	10.26	1.28 (1.04–1.59)	0.022		
Seasons								
Winter (Dec–Feb)	227/2,131	10.65	0.05	0.44	Ref.		617.46 (3)	<0.001
Summer (Mar–May)	652/2,120	30.75	2.26	7.36	3.73 (3.16–4.40)	<0.001		
Rainy (Jun–Aug)	978/2,195	44.56	5.60	12.58	6.74 (5.75–7.94)	<0.001		
Autumn (Sep–Nov)	751/2,204	34.07	4.04	11.85	4.34 (3.69–5.11)	<0.001		

CFR, case fatality rate; OR, odds ratio; CI, confidence interval.

**Table 3:** Association of non-modifiable host factors with prevalence, mortality, and case fatality of lumpy skin disease in cattle in northern Bangladesh

	Positive/total examined	Prevalence (%)	Mortality (%)	CFR (%)	OR (95% CI)	p-value	χ^2^-value (df)	p-value
Age								
Adult (>3 yr)	653/2,885	22.63	0.31	1.38	Ref.		156.86 (2)	<0.001
Calf (<1 yr)	1,188/3,177	37.39	5.98	15.99	2.04 (1.82–2.29)	<0.001		
Young (1–3 yr)	767/2,588	29.64	2.40	8.08	1.44 (1.28–1.63)	<0.001		
Breed								
Local	1,190/4,402	27.03	2.41	8.91	Ref.		74.47 (2)	<0.001
Friesian cross	547/1,895	28.87	2.74	9.51	1.10 (0.97–1.23)	0.136		
Sahiwal cross	871/2,353	37.02	4.38	11.83	1.59 (1.43–1.77)	<0.001		
Sex								
Male	1,170/4,032	29.02	2.73	9.40	Ref.		4.50 (1)	0.034
Female	1,438/4,618	31.14	3.27	10.50	1.11 (1.01–1.21)	0.032		

CFR, case fatality rate; OR, odds ratio; CI, confidence interval.

**Table 4:** Association of modifiable herd management and environmental factors with prevalence, mortality, and case fatality of lumpy skin disease in cattle in northern Bangladesh

	Positive/total examined	Prevalence (%)	Mortality (%)	CFR (%)	OR (95% CI)	p-value	χ^2^-value (df)	p-value
Vaccination								
Vaccinated	200/2,043	9.79	0.44	4.50	Ref.		525.25 (1)	<0.001
Non-vaccinated	2,408/6,607	36.45	3.81	10.47	5.28 (4.54–6.18)	<0.001		
Herd size								
Small (1–5 cattle)	563/2,185	25.77	2.06	7.99	Ref.		27.16 (2)	<0.001
Medium (6–10 cattle)	1,133/3,622	31.28	3.81	12.18	1.31 (1.16–1.48)	<0.001		
Large (11 or more cattle)	912/2,843	32.08	2.74	8.55	1.36 (1.20–1.54)	<0.001		
Production system								
Intensive	1,058/4,204	25.17	2.50	9.92	Ref.		96.01 (1)	<0.001
Semi-intensive	1,550/4,446	34.86	3.51	10.06	1.59 (1.45–1.75)	<0.001		
Biosecurity practice at herd								
Good	367/2,115	17.35	1.70	9.81	Ref.		246.70 (2)	<0.001
Moderate	1,148/3,637	31.56	2.25	7.14	2.20 (1.93–2.51)	<0.001		
Poor	1,093/2,898	37.72	4.93	13.08	2.88 (2.52–3.31)	<0.001		
Vector density at herd								
Low	373/2,411	15.47	1.16	7.51	Ref.		444.67 (2)	<0.001
Medium	952/3,170	30.03	2.59	8.61	2.35 (2.05–2.68)	<0.001		
High	1,283/3,069	41.81	4.92	11.77	3.93 (3.44–4.48)	<0.001		

CFR, case fatality rate; OR, odds ratio; CI, confidence interval.

**Table 5:** Univariate and multivariate logistic regression analysis of farm-level risk factors associated with lumpy skin disease occurrence in Bangladesh (n = 1,200)

Characteristic	LSD occurrence		Univariate logistic regression			Multivariate logistic regression		

	No (N = 584)	Yes (N = 616)	COR	95% CI	p-value	AOR	95% CI	p-value
District								
Bogura	62 (11%)	58 (9.4%)	Ref.	-		-	-	-
Dinajpur	58 (9.9%)	62 (10%)	1.14	0.69, 1.90	0.6	-	-	-
Gaibandha	63 (11%)	57 (9.3%)	0.97	0.58, 1.61	0.9	-	-	-
Joypurhat	59 (10%)	61 (9.9%)	1.11	0.67, 1.84	0.7	-	-	-
Kurigram	58 (9.9%)	62 (10%)	1.14	0.69, 1.90	0.6	-	-	-
Lalmonirhat	59 (10%)	61 (9.9%)	1.11	0.67, 1.84	0.7	-	-	-
Nilphamari	58 (9.9%)	62 (10%)	1.14	0.69, 1.90	0.6	-	-	-
Panchagarh	54 (9.2%)	66 (11%)	1.31	0.79, 2.17	0.3	-	-	-
Rangpur	61 (10%)	59 (9.6%)	1.03	0.62, 1.72	0.9	-	-	-
Thakurgaon	52 (8.9%)	68 (11%)	1.40	0.84, 2.33	0.2	-	-	-
Month								
April	49 (8.4%)	51 (8.3%)	Ref.	-		-	-	
August	34 (5.8%)	66 (11%)	1.87	1.06, 3.32	0.032	1.93	0.93, 4.07	0.080
December	65 (11%)	35 (5.7%)	0.52	0.29, 0.91	0.023	0.55	0.27, 1.12	0.10
February	78 (13%)	22 (3.6%)	0.27	0.14, 0.50	<0.001	0.32	0.15, 0.66	0.003
January	76 (13%)	24 (3.9%)	0.30	0.16, 0.55	<0.001	0.32	0.15, 0.67	0.003
July	32 (5.5%)	68 (11%)	2.04	1.15, 3.65	0.015	2.70	1.29, 5.71	0.009
June	30 (5.1%)	70 (11%)	2.24	1.26, 4.04	0.006	7.67	3.45, 17.4	<0.001
March	58 (9.9%)	42 (6.8%)	0.70	0.40, 1.21	0.2	0.51	0.26, 0.98	0.045
May	34 (5.8%)	66 (11%)	1.87	1.06, 3.32	0.032	1.53	0.77, 3.07	0.2
November	40 (6.8%)	60 (9.7%)	1.44	0.82, 2.53	0.2	2.45	1.21, 5.01	0.013
October	48 (8.2%)	52 (8.4%)	1.04	0.60, 1.81	0.9	0.99	0.50, 1.98	>0.9
September	40 (6.8%)	60 (9.7%)	1.44	0.82, 2.53	0.2	1.34	0.68, 2.64	0.4
Vaccination								
Yes	285 (49%)	56 (9.1%)	Ref.	-		-	-	
No	299 (51%)	560 (91%)	9.53	6.98, 13.2	<0.001	14.3	9.63, 21.7	<0.001
Herd size								
Small	297 (51%)	191 (31%)	Ref.	-		-	-	
Medium	216 (37%)	261 (42%)	1.88	1.46, 2.43	<0.001	1.76	1.28, 2.43	<0.001
Large	71 (12%)	164 (27%)	3.59	2.59, 5.03	<0.001	2.80	1.87, 4.22	<0.001
Production system								
Intensive	319 (55%)	278 (45%)	Ref.	-		-	-	
Semi-intensive	265 (45%)	338 (55%)	1.46	1.17, 1.84	0.001	0.85	0.61, 1.17	0.3
Biosecurity								
Good	208 (36%)	99 (16%)	Ref.	-		-	-	
Moderate	250 (43%)	269 (44%)	2.26	1.69, 3.04	<0.001	1.96	1.32, 2.92	<0.001
Poor	126 (22%)	248 (40%)	4.14	3.01, 5.72	<0.001	5.18	3.37, 8.08	<0.001
Vector density								
Low	219 (38%)	110 (18%)	Ref.	-		-	-	
Medium	222 (38%)	232 (38%)	2.08	1.55, 2.80	<0.001	1.51	1.00, 2.29	0.049
High	143 (24%)	274 (44%)	3.81	2.82, 5.19	<0.001	1.51	0.96, 2.40	0.077

p<0.05 was considered statistically significant.

LSD, lumpy skin disease; COR, crude odds ratio; CI, confidence interval; AOR, adjusted odds ratio.

## Data Availability

Upon reasonable request, the datasets of this study can be available from the corresponding author.

## SUPPLEMENTARY MATERIAL

**Supplement 1**. Month-wise prevalence of Lumpy Skin Disease (LSD) in the northern part of Bangladesh.
