# Supplementary material for: Epidemiology, risk factors, and spatio-temporal distribution of lumpy skin disease in cattle in northern Bangladesh
Source: Anim Biosci. 2026 Sep 16;39(11):260493. doi: 10.5713/ab.260493 (PMC13740646; doi:10.5713/ab.260493)
Supplement: Supplementary file 1 [file ab-260493-Supplementary-1.pdf]

Supplement 1. Month-wise prevalence of Lumpy Skin Disease (LSD) in northern part of Bangladesh.

**Months**	**Total cattle examined**	**Positive cases**	**Died**	**Prevalence (%)**	**Mortality (%)**	**Case fatality (%)**
September,23	393	158	21	40.20	5.34	13.29
October, 23	385	142	19	36.88	4.94	13.38
November, 23	370	104	13	28.11	3.51	12.50
December, 23	350	53	1	15.14	0.29	1.89
January, 24	356	33	0	9.27	0.00	0.00
February, 24	347	31	0	8.93	0.00	0.00
March, 24	356	93	2	26.12	0.56	2.15
April, 24	351	112	8	31.91	2.28	7.14
May, 24	357	127	16	35.57	4.48	12.60
June, 24	363	158	16	43.53	4.41	10.13
July, 24	362	164	23	45.30	6.35	14.02
August, 24	370	173	21	46.76	5.68	12.14
September, 24	355	134	10	37.75	5.92	15.67
October, 24	348	118	5	33.91	2.87	8.47
November, 24	353	95	0	26.91	1.42	5.26
December, 24	362	43	0	11.88	0.00	0.00
January, 25	360	39	0	10.83	0.00	0.00
February, 25	356	28	4	7.87	0.00	0.00
March, 25	353	89	5	25.21	1.13	4.49
April, 25	357	107	13	29.97	1.40	4.67
May, 25	346	124	20	35.84	3.76	10.48
June, 25	357	152	20	42.58	5.60	13.16
July, 25	367	163	23	44.41	5.45	12.27
August, 25	376	168	21	44.68	6.12	13.69
**24 Months**	**8650**	**2608**	**261**	**30.15**	**3.02**	**10.01**
